# Longitudinal Relationship of eHealth Literacy With Self-Management Via Self-efficacy in Individuals With Type 2 Diabetes: Autoregressive Cross-Lagged Modeling

**DOI:** 10.1097/jnr.0000000000000745

**Published:** 2026-05-08

**Authors:** Eun-Hyun LEE, Kwan-Woo LEE, Hae Jin KIM, Eun Hee KANG, Hyun Jung KANG

**Affiliations:** 1 Graduate School of Public Health, Ajou University, Suwon, Republic of Korea; 2 Department of Endocrinology and Metabolism, School of Medicine, Ajou University, Suwon, Republic of Korea; 3 Department of Nursing, Gangdong University, Eumseong-Gun, Republic of Korea; 4 College of Nursing, Ajou University, Suwon, Republic of Korea

**Keywords:** diabetes, eHealth literacy, longitudinal study, self-efficacy, self-management

## Abstract

**Background:**

In the internet era, eHealth literacy has become a recognized predictor of self-management in people with diabetes. Self-efficacy as a mediator of the relationship between eHealth literacy and self-management has been explored in cross-sectional studies only, which ignores its temporal sequence. Longitudinal studies on this mediated relationship in individuals with chronic diseases have not been previously conducted.

**Purpose:**

The purpose of this study was to explore the longitudinal relationship between eHealth literacy and self-management via self-efficacy in patients with type 2 diabetes.

**Methods:**

A prospective longitudinal survey was conducted using an autoregressive cross-lagged model. Measurements were made at three time points separated by 3-month intervals in 390 adults with type 2 diabetes at an outpatient clinic in a university hospital from May 2022 to January 2024 using a questionnaire consisting of several scales (i.e., the Condition-Specific eHealth Literacy Scale, Diabetes Management Self-Efficacy Scale, and Diabetes Self-Management Scale). The data were analyzed using structural equation modeling.

**Results:**

The autoregressive paths of all study variables were found to be significant across all time points, with eHealth literacy, self-efficacy, and self-management scores at each time point influencing their respective values at the subsequent time point (*β* = .95–.62, *p* < .001). Also, all of the cross-lagged paths were found to be significant across the investigated time points (*β* = .07–.21, *p* < .001). At the first time point, eHealth literacy was found to have a significant indirect effect on self-management at the third time point via self-efficacy at the second time point (bootstrap estimate *B* = 0.010, 95% confidence interval = [0.001, 0.028]).

**Conclusions/Implications for Practice:**

The results support that the longitudinal relationship between eHealth literacy and self-management is completely mediated by self-efficacy in people with type 2 diabetes. Health professionals should enhance the eHealth literacy of patients to boost their self-efficacy, making them better able to perform the regular tasks and behaviors required for diabetes self-management.

## Introduction

The term electronic health literacy (eHealth literacy: eHL), which first emerged in 2006, is defined as “the ability to seek find, understand, and appraise health information from electronic sources and apply the knowledge gained to addressing or solving a health problem” (Norman & Skinner, 2006). Research on eHL began in earnest after 2014 with the rapid development of internet technology and digital devices such as smartphones, tablets, and laptops (Wang et al., 2022).

There were 5.35 billion internet users worldwide in 2024, corresponding to ∼66.2% of the global population (We Are Social & DataReportal, 2024). Many people access the internet via digital devices to acquire health information and communicate with laypersons, health professionals, and institutes free of time and place limitations (Horigan et al., 2017). In terms of health information acquisition, most Koreans (56.8%) use the internet, followed by television (14.3%) and health professionals (9.5%; Bae & Kim, 2023). However, the high risk of misinformation/disinformation in data acquired via the internet demonstrates the importance of eHL, which describes the ability to acquire accurate health information from online sources such as search engines, social media platforms, and mobile apps (Swire-Thompson & Lazer, 2020). Thus, eHL has emerged as a predictor of the ability to improve health outcomes through activities such as disease self-management (Neter & Brainin, 2019).

Self-management is defined as “the individual’s ability to manage the symptoms, treatment, physical and psychosocial consequences and lifestyle changes inherent in living with a chronic condition” (Barlow et al., 2002). The number of adults living with diabetes worldwide (537 million in 2021) is expected to rise to 783 million by 2045 (International Diabetes Federation, 2021). For people with diabetes, self-management is essential to the effective control of blood glucose and the reduction of diabetic complications. eHL has been shown empirically to be a predictor of diabetes self-management efficacy (Guo et al., 2021). However, a systematic review of cross-sectional studies found only a weak direct correlation between eHL and disease management behaviors, with a coefficient of .24 (95% confidence interval [CI] = [0.12, 0.35]). However, this result should be interpreted cautiously due to the potential for mediating effects on this relationship (Kim et al., 2023).

Derived from social cognitive theory, self-efficacy refers to an efficacy expectation under which an individual believes that a particular action will produce a specific outcome (Bandura, 1977). Self-efficacy has previously been suggested in the literature to be a potential mediator between eHL and health outcomes (Xie et al., 2022). Lee et al. (2024) recently demonstrated eHL to be indirectly related to self-management through self-efficacy in patients with diabetes. Ji and Chi (2024) also reported eHL to influence self-care behaviors through self-efficacy. Similarly, self-efficacy has been reported to be a mediator in the relationship between eHL and self-management in people with chronic diseases (Wu et al., 2022). However, prior studies share a common limitation in terms of ignoring the temporal sequence of the mediation effect due to their use of a cross-sectional research design. Responding to this limitation, which increases the risk of bias (Maxwell & Cole, 2007), this study was designed to examine the issue using a longitudinal approach (Lee et al., 2024).

Cole and Maxwell (2003) noted that a mediation effect can only be tested properly in a longitudinal study. O’Laughlin et al. (2018) also emphasized the importance of applying longitudinal designs that reflect a temporal sequence in which an independent variable precedes a mediator, which in turn precedes a dependent variable.

To the best of the authors’ knowledge, no longitudinal study has been conducted to elucidate the mediating effect of self-efficacy on the relationship between eHL and self-management in people with chronic disease. Therefore, in this study, a longitudinal approach was used to clarify the temporal sequence of the mediation process in patients with type 2 diabetes. The hypothesis tested is that the longitudinal relationship of eHL with self-management is mediated via the role of self-efficacy in patients with type 2 diabetes.

## Methods

### Study Design

A prospective longitudinal survey was conducted to examine the relationship between eHL and self-management via self-efficacy. Testing a longitudinal mediation effect requires measurements to be made at three or more time points with an equal interval between any two adjacent time points to avoid biasing estimates of a mediating effect by failing to account for the previous values of a dependent variable (Cole & Maxwell, 2003). In a previous three-wave longitudinal study of the relationship between eHL and health outcome (including self-efficacy), a 3-month interval was used for measurements (Xie & Mo, 2024). A 3-month follow-up measure was also applied in a study of the effect of a mobile eHL program on patients with diabetes (Guo et al., 2023). Similarly, enhanced self-efficacy was found after a 12-week health literacy intervention in patients with diabetes (Lee et al., 2017). Based on these previous studies, three measurement time points separated by 3-month intervals were employed in this study.

### Study Participants

After approval of this study by the institutional review board of a hospital (AJIRB-MED-SUR-21-179), the study participants (*N* = 390) were recruited from May 2022 to January 2024 at a university hospital. The inclusions were: at least 19 years old, owning a smartphone, and having a diagnosis of type 2 diabetes. Based on the sample-size determination of a mediation analysis of longitudinal data (Pan et al., 2018), β_a_ (the relationship between the independent variable and mediating variable) and β_b_ (the relationship between the mediating variable and dependent variable after controlling for the effects of the independent variable) values of .14, .26, .39, and .59 were defined in this study as small, moderate, halfway (between moderate and large), and large effect sizes, respectively. In a previous cross-sectional study, the respective β_a_ (the relationship between eHL and self-efficacy) and β_b_ (the relationship between self-efficacy and self-management after controlling for the effect of eHL) values used were ∼.34 and .76 (Lee et al., 2024). Based on these effect size values, to achieve 80% statistical power for three measurement time points, a minimum of 148 participants were estimated to be needed for the last measurement (Pan et al., 2018). Because a longitudinal survey approach that collected data via the public postal system was used in this study, a lower response rate (as low as 26%; Larson & Poist, 2004) was anticipated. Thus, a sample size of 390 was recruited for the first measurement time point.

### Measurements

The main variables related to eHealth literacy, self-efficacy, and diabetes self-management in this study were respectively measured using the Condition-Specific eHealth Literacy Scale for Diabetes (CeHLS-D; Lee et al., 2022), the Korean version of the Diabetes Management Self-Efficacy Scale (DMSES; Lee et al., 2015), and the Diabetes Self-Management Scale (DSMS; Lee et al., 2020).

#### eHealth literacy

eHL was measured using the CeHLS-D, which comprises the two subscales of cognitive actions for internet diabetes information (seven items) and digital communication ability (three items; Lee et al., 2022). Each item is scored on a 5-point Likert scale, with higher scores representing better eHL. Total scale and subscale scores are calculated as the respective average of each total and subscale item. In the original study in which the CeHLS-D was proposed, the following psychometric properties were satisfied in 453 patients with diabetes: content validity, structural validity, convergent validity, known-groups validity, internal consistency (Cronbach’s alpha for the subscales ranged from .92 to .89), and measurement invariance across gender, age, and glycemic control groups. The Cronbach’s alpha for the total/subscales of the CeHLS-D at T1, T2, and T3 in this study ranged from .78 to .92.

#### Self-efficacy

Self-efficacy was measured using the Korean version of the DMSES (Lee et al., 2015). This scale comprises 16 items scored on an 11-point scale, with higher scores implying better diabetes self-management self-efficacy. The DMSES has four subscales covering nutrition (6 items), physical exercise/body weight (4 items), medical treatment (3 items), and blood glucose (3 items), with sums totaled to derive the total and subscale scores. This scale was previously shown to have satisfactory psychometric properties in 440 people with diabetes in terms of content validity, structural validity, concurrent validity, internal consistency (Cronbach’s alpha for subscales = .84–.89) and a test–retest reliability (intraclass correlation coefficient) of .85. The Cronbach’s alpha for the total/subscale scores of the DMSES at T1, T2, and T3 ranged from .75 to .91 in this study.

#### Diabetes self-management

The DSMS comprises 17 items in the following subscales: physical exercise (4 items), diet (2 items), stress alleviation (2 items), taking medication (2 items), self-monitoring of blood glucose (3 items), and self-regulation (4 items; Lee et al., 2020). Each item is scored on a 5-point Likert scale, with higher scores representing better self-management. Total and subscale scores are calculated as the summed average of item scores. In the original study in which the DSMS was proposed, the following psychometric properties were satisfactory in 472 people with diabetes: content validity, structural validity, convergent validity, internal consistency (Cronbach’s alpha values = .76–.84), and test–retest reliability (intraclass correlation coefficient = .84–.92). The Cronbach’s alpha for the total/subscales of the DSMS at T1, T2, and T3 ranged from .74 to .93 in this study.

#### General information of participants

Information on participant age, gender, education, marital status, and income was collected in this study using a self-report survey at T1. Also, information regarding treatment regimens, duration of diabetes diagnosis, and glycated hemoglobin (HbA_1_c) levels was collected from medical records.

### Data Collection

Two trained research assistants met potential participants at an outpatient clinic, providing them with information about this study. Those who agreed to participate were asked to sign an informed-consent form. Data were collected at 3-month intervals using a questionnaire consisting of the three above-described scales. The first set of data was collected (first time point: T1) on the day each participant signed informed consent at the outpatient clinic. The second and third sets of data (second and third time points: T2 and T3) were provided from home by the participants and mailed to the researchers via the public post in a pre-stamped and addressed return envelope.

### Data Analyses

Data were analyzed using SPSS version 29 and AMOS version 25 (IBM Corp., Armonk, NY, USA). Descriptive statistics, Pearson’s correlation coefficients, and Cronbach’s alpha values were calculated using SPSS, while structural equation modeling (SEM) was conducted using AMOS.

Within the SEM framework, longitudinal mediation analyses may be conducted using the autoregressive cross-lagged model (ACLM), latent growth-curve model, or latent difference-score model, with the appropriate analysis method selected based on the research question of interest. When investigating a causal mediation effect among variables measured over time, ACLM is the most appropriate model, and when changes in means over time are of interest, latent growth-curve and latent difference-score models are preferable (O’Laughlin et al., 2018). ACLMs may be classified into standard and random-intercept ACLMs. For research designed to confirm the directionality of a mediation association, standard ACLM is sufficient (Baribeau et al., 2022). Thus, ACLM was employed in this study.

**Figure 1:**
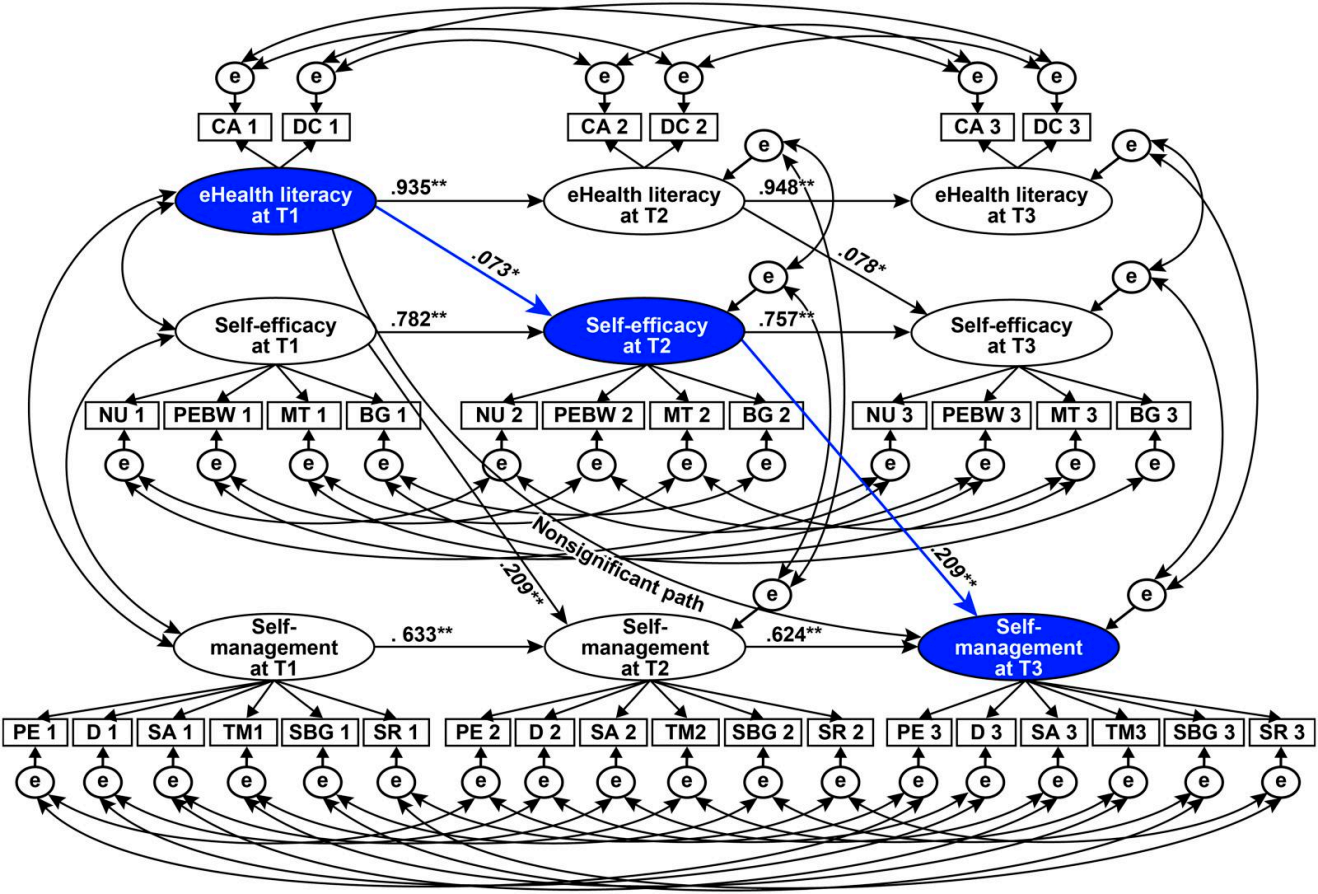
*Autoregressive Cross-Lagged Model for the Longitudinal Relationship of eHealth Literacy with Self-Management Via Self-Efficacy Note*. Path coefficients are standardized estimates. The values of factor loadings (.452–.990) and error covariances between latent variables (.189–.557) are not depicted in order to reduce figure complexity. e = measurement error; T1 = first time point; T2 = second time point; T3 = third time point. *p = .008, **p < .001. Indicators of latent variables (eHealth literacy, self-efficacy, and self-management): CA (cognitive actions for internet diabetes information); DC (digital communication ability); NU (nutrition); PEBW (physical exercise/body weight); MT (medical treatment); BG (blood glucose); PE (physical exercise); D (diet); SA (stress alleviation); TM (taking medication); SBG (self-monitoring of blood glucose); and SR (self-regulation).

The longitudinal mediation model used in this study is shown in Figure 1. SEM with maximum likelihood was used to test the ACLM. Data outliers were assessed using Mahalanobis *d*-squared values, and the farthest case from the centroid at *p* < .001 were deleted (Byrne, 2016). Normality was determined when the absolute skewness value was ≤ 2, and the absolute (excess) kurtosis value was ≤ 4, as suggested by Kim (2013) for sample sizes > 300. The measurement invariance, structural (autoregressive and cross-legged) path invariance, and error covariance invariance of the study model were then assessed across time points in hierarchical order to assess the assumptions of the ACLM: no constraints (Model 1), constraints on factor loadings (Models 2–4), constraints on autoregressive paths (Models 5–7), constraints on cross-lagged paths (Models 8 and 9), and constraints on error covariance (Models 10–12). The criteria used to assess goodness of fit included: a comparative fit index (CFI) of > .90, a Tucker-Lewis index (TLI) of > .90, and a root-mean-square error of approximation (RMSEA) of < .80 (Little, 2024). By adding invariance constraints into the hierarchical order, the fit indices of the nested models were compared with the criteria for changes in CFI (∆CFI) of < .010, in TLI (∆TLI) of < .010, and in RMSEA (∆RMSEA) of < .015 (Chen, 2007). Finally, the statistical estimate for the longitudinal mediation effect was examined using a 95% bias-corrected bootstrap CI with 5,000 resamples, with a value not including zero indicating a significant indirect effect.

## Results

### General Characteristics

The first time point (T1) included 390 participants, with 46 and 13 dropping out at the second (T2) and third (T3) time points, respectively. Thus, data from 331 participants were available across all three measurement time points and included in the final analysis. The general characteristics of the 331 participants are presented in Table 1. More than half were male (67.7%), married or living together (83.1%), and held a bachelor’s degree or higher (57.4%). The average age of the sample was 54.1 years (*SD* = 10.1). Approximately three-quarters were taking an oral hypoglycemic agent (75.8%) and had uncontrolled HbA_1_c levels (72.2%). The average duration of diabetes in the sample was 11.0 years (*SD* = 7.8).

**Table 1:** General Participant Characteristics at Baseline (*N* = 331)

Variable	*n* (%)
Sex	
Male	224 (67.7)
Female	107 (32.3)
Age (years, *M* and *SD*)	
Educational level	54.1±10.1
Middle school or below	24 (7.2)
High school	113 (34.1)
Bachelor’s degree or above	190 (57.4)
Others	4 (1.2)
Marital status	
Married or cohabitating	275 (83.1)
Never married single	35 (10.6)
Divorced or widowed	19 (5.7)
Others	2 (0.6)
Monthly income (KRW)	
< 3,000,000	97 (29.3)
3,000,000–3,999,999	70 (21.1)
≥ 4,000,000	164 (49.5)
Treatment regimen	
Oral hypoglycemic agent	251 (75.8)
Insulin	7 (2.1)
Oral hypoglycemic agent + insulin	73 (22.1)
Duration of disease (years, *M* and *SD*)	11.0±7.8
HbA1c level	
Controlled (< 6.5%)	92 (27.8)
Uncontrolled (≥ 6.5%)	239 (72.2)

*Note*. KRW = South Korean won; HbA1c = glycated hemoglobin.

### Descriptive Statistics and Correlations Among Study Variables

The missing value percentage among all item responses was 0.46. As a missing-data rate of < 5% is considered negligible, the missing data could be handled using either the deletion or likelihood method (Mirzaei et al., 2022). In this study, an expectation-maximization algorithm was used as the likelihood method to deal with the missing data. Floor and ceiling effects on the scores for each instrument were explored. When 15% or more respondents achieve the lowest or highest scores on an instrument, these may be interpreted as, respectively, the floor or ceiling effects (McHorney & Tarlov, 1995). In this study, no floor or ceiling effects were found for the eHL, self-efficacy, or self-management scores at any of the time points. The descriptive statistics and coefficients for the correlations among eHL, self-efficacy, and self-management at the three time points are presented in Table 2. All of the intercorrelations were significant.

**Table 2:** Mean and Standard Deviation Scores of the Study Variables, and Coefficients for Their Correlations

Variable	Mean (*SD*)	1	2	3	4	5	6	7	8	9
1. eHealth literacy at T1	2.81 (0.79)									
2. eHealth literacy at T2	2.91 (0.79)	.80**								
3. eHealth literacy at T3	3.00 (0.79)	.79**	.88**							
4. Self-efficacy at T1	111.33 (26.65)	.44**	.47**	.41**						
5. Self-efficacy at T2	112.44 (25.45)	.35**	.44**	.39**	.72**					
6. Self-efficacy at T3	113.49 (24.24)	.37**	.46**	.45**	.75**	.79**				
7. Self-management at T1	2.30 (0.62)	.21**	.22**	.15*	.60**	.51**	.49**			
8. Self-management at T2	2.33 (0.59)	.19**	.25**	.18**	.56**	.60**	.55**	.73**		
9. Self-management at T3	2.34 (0.59)	.21**	.26**	.22**	.52**	.53**	.60**	.70**	.77**	

*Note*. eHealth literacy = electronic health literacy. T1 = first time point; T2 = second time point; T3 = third time point.

*p < .05. **p < .001.

### Model Invariance Across the Three Time Points

Data were screened for use in the SEM analysis. Based on the Mahalanobis *d*-squared values, for which *p* < .001, the 15 outliers were deleted. The remaining data were consistent with the skewness and kurtosis criteria, implying normal distributions (Table 3). Model fit statistics for the baseline, measurement invariance, structural path invariance, and error covariance invariance over the three time points are presented in Table 4. The no-constraints baseline model (Model 1) provided a satisfactory goodness of fit (CFI = .932, TLI = .921, and RMSEA = .059), implying the model adequately represented the data. This was also the case for Model 2 (constraints on the factor loadings of eHL over the time points), with CFI = .932, TLI = .921, and RMSEA = .059. The differences among goodness-of-fit values (compared with those in Model 1) were also consistent with the criteria for invariances of ∆CFI < .010, ∆TLI < .010, and ∆RMSEA < .015. Moreover, the remaining models all exhibited a satisfactory goodness-of-fit, and their nested model comparisons for invariances were all satisfactory. Therefore, all of the invariance assumptions for the ACLM used in this longitudinal mediation study were satisfied, and Model 12 was selected as the final model.

**Table 3:** Skewness and Kurtosis Values of the Observed Variables

Variable	Skewness	Kurtosis
Self-regulation at T3	–0.312	–0.441
Self-monitoring of blood glucose at T3	0.428	–0.656
Taking medication at T3	–0.866	0.516
Stress alleviation at T3	–0.280	–0.238
Diet at T3	–0.274	–0.147
Physical exercise at T3	0.239	–0.515
Self-regulation at T2	–0.210	–0.684
Self-monitoring of blood glucose at T2	0.399	–0.727
Taking medication at T2	–1.052	1.230
Stress alleviation at T2	–0.286	0.012
Diet at T2	–0.245	–0.087
Physical exercise at T2	0.083	–0.711
Self-regulation at T1	–0.014	–0.591
Self-monitoring of blood glucose at T1	0.365	–0.865
Taking medication at T1	–0.878	0.242
Stress alleviation at T1	–0.293	–0.405
Diet at T1	–0.152	–0.304
Physical exercise at T1	0.158	–0.557
Blood glucose at T3	–1.048	1.015
Medical treatment at T3	–1.709	3.574
Physical exercise/body weight at T3	–0.512	–0.167
Nutrition at T3	–0.319	0.027
Blood glucose at T2	–1.033	0.736
Medical treatment at T2	–1.600	2.701
Physical exercise/body weight at T2	–0.310	–0.630
Nutrition at T2	–0.100	–0.520
Blood glucose at T1	–1.075	0.779
Medical treatment at T1	–1.594	2.709
Physical exercise/body weight at T1	–0.437	–0.242
Nutrition at T1	–0.255	–0.038
Digital communication ability at T3	–0.965	–0.126
Cognitive actions for internet diabetes information at T3	–0.733	1.136
Digital communication ability at T2	–0.926	–0.294
Cognitive actions for internet diabetes information at T2	–0.515	0.459
Digital communication ability at T1	–0.883	–0.015
Cognitive actions for internet diabetes information at T1	–0.578	1.009

*Note*. T3 = third time point; T2 = second time point; T1 = first time point.

**Table 4:** Model Fit Statistics in Invariance Comparison Tests

Invariance Model	χ^2^	*df*	CFI	TLI	RMSEA	∆CFI	∆TLI	∆RMSEA
Baseline								
1	1130.38**	540	.932	.921	.059			
Measurement invariance								
2	1132.36**	542	.932	.921	.059	< .001	< .001	< .001
3	1148.11**	548	.931	.921	.059	–.001	< .001	< .001
4	1158.93**	558	.931	.922	.058	< .001	.001	–.001
Autoregressive invariance								
5	1159.11**	559	.931	.922	.058	< .001	< .001	< .001
6	1159.23**	560	.931	.923	.058	< .001	.001	< .001
7	1161.20**	561	.931	.923	.058	< .001	< .001	< .001
Cross-lagged invariance								
8	1174.88**	562	.930	.921	.059	–.001	–.002	.001
9	1175.04**	563	.930	.922	.059	< .001	.001	< .001
Error covariance invariance								
10	1178.04**	564	.931	.921	.059	.001	–.001	< .001
11	1178.07**	565	.930	.922	.059	–.001	.001	< .001
12	1178.18**	566	.930	.922	.059	< .001	< .001	< .001

*Note.* Constraints on the models were progressively added in hierarchical order: Model 1, baseline model (no constraints); Model 2, constraints on factor loadings of eHealth literacy; Model 3, constraints on factor loadings of self-efficacy; Model 4, constraints on factor loadings of self-management; Model 5, constraints on autoregressive paths of eHealth literacy; Model 6, constraints on autoregressive paths of self-efficacy; Model 7, constraints on autoregressive paths of self-management; Model 8, constraints on cross-lagged path from eHealth literacy to self-efficacy; Model 9, constraints on cross-lagged path from self-efficacy to self-management; Model 10, constraints on error covariance between eHealth literacy and self-efficacy; Model 11, constraints on error covariance between self-efficacy and self-management; Model 12, constraints on error covariance between eHealth literacy and self-management. CFI = comparative fit index; TLI = Tucker-Lewis index; RMSEA = root-mean-square error of approximation; ∆ = difference between index values in a nested model comparison.

**p < .001.

### Longitudinal Mediation Analysis

The results from the study using Model 12 are presented in Figure 1. The autoregressive paths of each study variable were all determined to be statistically significant across the time points, with each eHL, self-efficacy, and self-management value at the prior time point influencing its value at the subsequent time point. Also, all of the cross-lagged paths were confirmed to be statistically significant across time points. The direct path of eHL at T1 to self-management at T3 failed to meet statistical significance. The eHL at T1 was shown to have a statistically significant indirect effect on self-management at T3 via self-efficacy at T2 in the bootstrapping analysis (bootstrap estimate *B* = 0.010, 95% CI = [.001, .028], *p* = .019), indicating the relationship was fully mediated by self-efficacy in the temporal sequence.

## Discussion

### Main Findings

The findings of this study support the hypothesized longitudinal relationship between eHL and self-management via self-efficacy in patients with type 2 diabetes. As the first longitudinal study on the mediating role of self-efficacy in this relationship, comparing its findings with those of previous cross-sectional studies is worthwhile. In this study, eHL at T1 was found to relate directly to self-efficacy at T2, while self-efficacy at T2 was also found to relate directly to self-management at T3. These findings are consistent with a previous empirical finding in a cross-sectional study of a relationship between eHL and self-efficacy in patients with chronic disease (Wu et al., 2022) and a prior systematic review finding that self-efficacy affects self-management significantly in adults (Qin et al., 2020).

In this study, eHL at T1 was shown to relate indirectly to self-management at T3, while the direct relationship between eHL at T1 and self-management at T3 was not found to reach statistical significance. These findings indicate that self-efficacy completely mediated the causal influence of eHL on self-management. This is consistent with a previous cross-sectional study finding that self-efficacy fully mediated the relationship between eHL and self-management in patients with diabetes (Lee et al., 2024). Similarly, self-efficacy was found to partially mediate the relationship in people with chronic noncommunicable diseases in a cross-sectional study (Wu et al., 2022) that measured eHL using the eHealth Literacy Scale (eHEALS; Norman & Skinner, 2006). According to a systematic review of the psychometric properties of eHL instruments, the eHEALS does not sufficiently measure the comprehensive skills needed to address the dynamic and social nature of eHL because it was developed during the first generation of simple health information technology (aka Web 1.0; Lee et al., 2021). Thus, future studies on this issue should use the eHL scale, which takes into consideration the nature of the current social media environment.

Together, the findings of this study support that self-efficacy mediates the relationship between eHL and self-management in individuals with diabetes living in the current eHealth era. More specifically, patients with higher levels of eHL were found to be more likely to obtain accurate information about diabetes self-management from electronic sources and to interact regularly with others on the internet and, as a result, were more likely to have higher self-efficacy in terms of viewing diabetes self-management behaviors as challenges to be overcome. Moreover, those with higher levels of self-efficacy were identified as more likely to successfully perform beneficial tasks and behaviors such as physical exercise, diet, stress control, medication adherence, blood glucose self-monitoring, and foot care.

### Implications for Practice

The results of this longitudinal study provide empirical evidence for the mechanism underlying the linkage of eHL to diabetes self-management through self-efficacy. This finding may assist health professionals in developing more effective methods of raising patient self-management capabilities in clinical practice. To improve the self-management efficacy of patients with diabetes living in the current internet era, health professionals should apply interventions that include strategies aimed at enhancing both eHL and self-efficacy abilities by implementing strategies to enhance the former first and the latter afterward. Although, to the best of the authors’ knowledge, this integrated intervention approach does not exist, components of this approach have been previously reported. For example, Chang et al. (2022) developed an eHL intervention based on information-motivation-behavioral theory (Fisher et al., 2003), and found a significant improvement in eHL scores in older adults using a single-group, pretest and posttest study design. Similarly, Guo et al. (2023) developed an educational mobile eHL program for patients with diabetes and found significantly improved eHL abilities at 3-month follow-up in a pretest and posttest experimental study. Social cognitive theory (Bandura, 1977) has primarily been applied as the framework for self-efficacy programs. Van de Laar and van der Bijl (2001) suggested strategies for enhancing self-efficacy in patients with diabetes using four sources of information based on that theory, namely performance accomplishments, vicarious experiences, verbal persuasion, and physiological/emotional arousal. Self-efficacy-focused programs such as those have empirically exhibited an effect on self-management in patients with type 2 diabetes up to 12-month follow-up, with the effect peaking at 3–6 months post-intervention (Jiang et al., 2019; Jiang et al., 2021). Therefore, health professionals should be encouraged to adopt and integrate the above-described types of interventions into their clinical practice to enhance eHL and self-efficacy to improve self-management efficacy in patients with diabetes.

### Implications for Research

Identifying potential relationship mediators is a major issue associated with the relationship between eHL and health outcomes (Xie et al., 2022). Empowerment and psychological distress have, respectively, previously been proposed as potential mediators of this relationship in individuals with diabetes and heart failure (Lee et al., 2024; Lin et al., 2020). In addition, empirical evidence for empowerment completely mediating the relationship between eHL and self-management in patients with chronic kidney disease was found in a recent cross-sectional study (Shiu et al., 2023). In light of these findings, longitudinal mediation studies designed to investigate empowerment and psychological distress as potential mediators of the relationship in patients with diabetes are recommended. Furthermore, a longitudinal mediation study designed to consider multiple mediators of self-efficacy, empowerment, and psychological distress together is recommended.

### Strengths and Limitations

This study has several major strengths. First, this was the first longitudinal mediation study conducted to assess the relationship between eHL and self-management via the mediator of self-efficacy, with an indirect relationship allowed in a temporal sequence. Second, eHL was measured using the CeHLS-D, which is a psychometrically satisfactory diabetes-specific instrument designed to account for the influence of current social media habits on eHealth. In studies using either multiple regression or path analysis, composite scores (e.g., total mean or sum scores) are commonly used to represent independent, mediation, and dependent variables based on the assumption of no measurement errors in the scores, which often introduces bias into mediation effect estimates (Cole & Maxwell, 2003). Third, SEM was applied and latent variables were measured using multiple indicators to account for measurement errors.

This study was also affected by several limitations. First, the measurements were made at three time points separated by 3-month intervals, as at least three time points are necessary for a valid longitudinal mediation analysis. Thus, determining the duration of the mediation effect of self-efficacy beyond these three measurement time points was not possible. Second, this study was conducted in a country with an internet penetration rate of 97.0% and a smartphone penetration rate among adults of 95% (Kemp, 2021; Silver, 2019). Thus, replication research will be necessary in other countries with a sufficiently wide range of digital environments to confirm the generalizability of the findings.

### Conclusions

This was the first longitudinal mediation study to examine the relationship between eHL and self-management through the mediator of self-efficacy. Successive measurements were made at three time points separated by 3-month intervals. Based on the findings, self-efficacy completely mediates the causal influence of eHL on self-management in patients with type 2 diabetes. In the eHealth era, health professionals must enhance eHL in patients to effectively improve their self-efficacy and help ensure they have the requisite knowledge and abilities to perform diabetes self-management tasks and behaviors on their own in their daily lives.
